# Ethics and Algorithms to Navigate AI’s Emerging Role in Organ Transplantation

**DOI:** 10.3390/jcm14082775

**Published:** 2025-04-17

**Authors:** Amankeldi A. Salybekov, Ainur Yerkos, Martin Sedlmayr, Markus Wolfien

**Affiliations:** 1 Regenerative Medicine Division, Cell and Gene Therapy Department, Qazaq Institute of Innovative Medicine, Astana 020000, Kazakhstan; 2 Department of Computer Science, Al-Farabi Kazakh National University, Almaty 050040, Kazakhstan; yerkosova@gmail.com; 3 Institute for Medical Informatics and Biometry, Faculty of Medicine Carl Gustav Carus, TUD Dresden University of Technology, 01069 Dresden, Germany; martin.sedlmayr@tu-dresden.de; 4 Center for Scalable Data Analytics and Artificial Intelligence (ScaDS.AI), 01069 Dresden, Germany

**Keywords:** artificial intelligence, solid organ transplantation, graft failure, machine learning, deep learning

## Abstract

**Background/Objectives**: Solid organ transplantation remains a critical life-saving treatment for end-stage organ failure, yet it faces persistent challenges, such as organ scarcity, graft rejection, and postoperative complications. Artificial intelligence (AI) has the potential to address these challenges by revolutionizing transplantation practices. **Methods**: This review article explores the diverse applications of AI in solid organ transplantation, focusing on its impact on diagnostics, treatment, and the evolving market landscape. We discuss how machine learning, deep learning, and generative AI are harnessing vast datasets to predict transplant outcomes, personalized immunosuppressive regimens, and optimize patient selection. Additionally, we examine the ethical implications of AI in transplantation and highlight promising AI-driven innovations nearing FDA evaluation. **Results**: AI improves organ allocation processes, refines predictions for transplant outcomes, and enables tailored immunosuppressive regimens. These advancements contribute to better patient selection and enhance overall transplant success rates. **Conclusions**: By bridging the gap in organ availability and improving long-term transplant success, AI holds promise to significantly advance the field of solid organ transplantation.

## 1. Introduction

According to global transplantation and donation databases, as well as international registries in organ transplantation reports, Western countries are leading in solid organ transplantation per million population in 2023 [1,2]. Every donor has the potential to save at least eight lives and enhance over 75 others [3]. The United States, for example, is among the top in transplantation rates, with over 46,000 transplants performed, including those from deceased and living donors [3]. However, a recent report by the United Network for Organ Sharing (UNOS) revealed a steady increase in the number of patients on the waiting list, which, as of March 2024, included more than 103,000 patients [3]. Despite the high transplantation rate and a three-tiered system designed to ensure more equitable organ allocation, the demand for transplanted organs currently exceeds the supply, with 17 people dying each day while waiting for a transplant in the USA [3]. This situation highlights the need for future improvements in solid organ transplantation. Consequently, to enhance the allocation and transplantation process, artificial intelligence (AI) has recently been introduced [4,5,6,7].

The accessibility of healthcare data and advancements in analytical methods have driven the exponential growth of AI across various medical fields. AI algorithms can create models that automatically learn and generate predictions based on previous knowledge or experience, improving information processing without needing explicit programming for all possible cases. Furthermore, many AI algorithms can improve over time, adapting to new data and resulting in continuous system enhancement and increased accuracy. AI significantly enhances the complex decision-making involved in pre- and post-transplant patient care by analyzing vast datasets and offering clinical recommendations [8]. Notably, AI-based classifiers are being used to improve organ allocation and donor-recipient matching [7], immunosuppression management in real-time [9], and precision in transplant pathology [10,11]. The goal of AI is to uncover hidden patterns and intricate relationships in large datasets, enabling logical outcomes and efficient resource use. Although still in the early stages and lacking validated algorithms for precise organ selection, rejection prediction, or reduction of postoperative complications, AI has already made strides in reducing rejection rates and improving transplantation and organ preservation techniques over recent decades. As AI continues to advance, it holds the potential to transform not only the technical aspects of transplantation but also the ethical landscape for patients and physicians. The integration of AI in clinical workflows introduces new ethical considerations, such as ensuring transparency in AI-driven decisions, maintaining patient autonomy, and addressing potential biases in algorithmic predictions. For physicians, the advent of AI-enhanced decision-making tools, particularly those that may receive FDA approval in the near future, could necessitate updates to clinical workflows and protocols. These tools, while promising in their ability to improve outcomes and streamline processes, also require careful consideration of the physician’s role in interpreting AI recommendations and maintaining the patient–physician relationship. This review explores the current and potential future applications of AI in solid organ transplantation, while also examining the ethical challenges and changes in clinical practice that may arise as AI-based systems become more integrated into the field.

## 2. Materials and Methods

A literature search was conducted in the PubMed, Google Scholar, Cochrane, and Scopus databases using the keywords “artificial intelligence”, “artificial intelligence in solid organ transplantation”, “artificial intelligence guidelines”, and “artificial intelligence in transplantation”. Additionally, reference lists from relevant studies were manually reviewed. Only English-language guidelines, research articles, and reviews were selected for inclusion. The exclusion criteria were as follows:

1. Studies not written in English;
2. Conference abstracts, notes, letters, case reports, or animal studies;
3. Duplicate studies.

## 3. Evolution of Data Analysis—From Traditional Statistics to Generative AI

**Figure 1:**
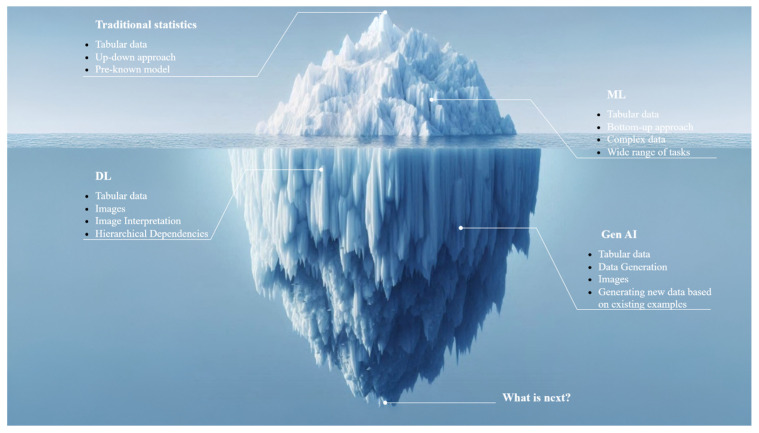
Transformation of data analysis strategies. Traditional statistics are ideal for evaluating small sample tabular data, while machine learning algorithms are suited for more complex datasets and perform best when trained on large amounts of data. Deep learning allows for the analysis of both medical images and tabular data, but unlike Generative AI, it cannot produce new data.

Recent years have witnessed rapid growth in research on AI and its applications in healthcare, driven by advancements in computing technology and a surge in the volume of digital data available for analysis. Additionally, the increasing complexity of medical data, such as genetic, follow-up, imaging, and laboratory data, often renders traditional statistical methods insufficient for accurate interpretation. Consequently, new AI-based research has demonstrated that AI-assisted analysis of donor and recipient data can enhance the prediction of short- and long-term solid organ transplant survival [12]. Figure 1 presents the transformation of data analysis strategies. Statistical methods are effective tools for analyzing data across various fields, including medicine. Traditional statistical methods are commonly employed to identify relationships between input and output data. However, there are notable differences between classical statistical learning and modern machine learning methods, primarily concerning their capabilities and limitations. Traditional statistical methods, such as linear and logistic regression, follow a top-down approach. In this approach, the researcher starts with a known model and estimates its parameters based on the available data [13]. Traditional statistical methods are effective for analyzing datasets with limited size and complexity. However, their capabilities diminish as the volume and diversity of information increase. In such scenarios, it is advisable to employ AI methods, such as machine and deep learning, which offer greater flexibility and can solve a wider range of problems [14]. These methods follow a bottom-up approach, starting with the data and allowing the algorithm to develop a model with the primary goal of prediction [13]. The resulting models are often complex, and some parameters cannot be directly estimated from the data. Instead, these parameters are either selected based on relevant previous studies or manually tuned during training to provide the best prediction. Compared to traditional statistical methods, machine learning algorithms can handle a larger number of complex variables—ordinal, nominal, etc.—but they also require a larger sample size for analysis. As a result, while machine learning algorithms can deliver remarkable results in certain contexts, their effectiveness depends on the quality and quantity of available data, as well as the specifics of the task [15,16]. For example, it can be argued that machine learning does not outperform Cox proportional hazards models in specific cases [17]. To address this issue, it is essential to understand that all machine learning algorithms have their own advantages and limitations and may not be suitable for all given tasks. In other words, machine learning can handle complex interactions in large datasets to more accurately predict outcomes, but these models require a larger number of input and output pairs for training, which traditional statistical methods may not require.

Deep learning and generative AI are two powerful subsets of machine learning, each offering unique advantages in the field of organ transplantation. Deep learning, characterized by its use of layered neural networks, excels in analyzing and interpreting complex medical data, such as images from CT scans or biopsy results. This capacity enables deep learning models to assess the suitability of organs for transplant and predict patient outcomes with high accuracy by capturing intricate patterns and dependencies in imaging data that traditional models might overlook. On the other hand, generative AI focuses on synthesizing new data points, which can be instrumental in organ transplantation for creating realistic, anonymized medical datasets for images but also for tabular data [18,19]. These synthesized datasets can be used for training more robust models without the privacy concerns associated with using real patient data [20]. Generative AI can also simulate various scenarios of organ matching, helping to optimize the allocation and expected outcomes of organ transplants. Additionally, generative AI, by creating new content based on training data, has the potential to greatly simplify tasks in transplantation, from research and education to clinical practice and patient education, provided that it is continually improved and implementation challenges are addressed [21]. The combination of these two approaches aims to transform transplantation by improving predictive models, optimizing organ selection, and improving the prognostic tools available to physicians, which in turn increases transplant success rates and improves patient care. However, to fully realize the potential of AI to improve the lives of patients in need of transplantation, it is necessary to ensure that AI systems are transparent and understandable and to provide physicians with detailed explanations of how they work and are validated [22].

As these AI-based innovations mature, we are getting closer to having advanced tools specifically designed for kidney, heart, and liver transplantation. These tools, some of which are already in the FDA approval process, promise to improve every aspect of transplantation, as will be detailed in the following sections.

## 4. AI Tools in Transplantation

**Figure 2:**
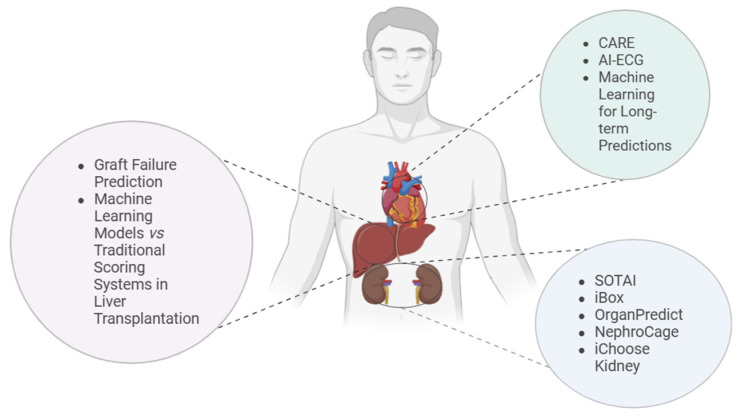
AI tools for organ transplantation. The illustration shows the anatomical locations of the human kidney, heart, and liver, followed by a list of AI-based tools used in the transplantation of each organ. On the left are the kidney transplant tools, including iBox, SOTAI, OrganPredict, NephroCage, and iChoose Kidney. In the center, above the heart, are the heart transplant tools, including CARE, AI-ECG, and machine learning models for long-term prediction. On the right, above the liver, are the liver transplant tools, including graft rejection prediction and a comparison of machine learning models with traditional scoring systems.

Artificial intelligence (AI) is increasingly being used in various fields of medicine, including organ transplantation, to improve treatment efficiency and optimize clinical decision-making. Figure 2 shows some of the AI-based tools used in kidney, heart, and liver transplantation.

### 4.1. Kidney Transplantation

In the field of kidney transplantation, AI-based tools have been developed both in academic and commercial settings to predict outcomes, such as graft survival and immunosuppression, and to improve donor–recipient matching [9,22,23,24]. Here, we discuss prominent AI-based tools in kidney transplantation.

iBox: This tool was developed by an international team led by French researchers and is designed to predict the risk of kidney transplant loss [13,25]. The features include estimated glomerular filtration rate (eGFR), proteinuria, anti-human leukocyte antigen donor-specific antibody, and kidney graft biopsy histopathology, which can be assessed at any time after the transplant. iBox has been validated and tested on over 7500 patients with a combination of the above-mentioned parameters to provide personalized predictions of patient outcomes three, five, and ten years after the transplant [13,25]. Initially developed for individual patient use to guide clinical care and management of kidney transplant recipients, iBox was later adapted in collaboration with the Paris Transplant Group, Critical Path Institute, and other transplant community members to create the Transplant Therapeutics Consortium (TTC) for clinical trials [14,26]. This adaptation, approved by the European Medicines Agency (EMA) [19,27], aims to enhance drug development by using early data to predict long-term results. Five datasets supporting the regulatory endorsement of iBox, representing over 2500 de novo kidney transplant recipients with 1-year iBox assessments, were evaluated [18,28]. Furthermore, these datasets included details about each component measured by the iBox assay and documentation certifying that the analytical methods were strong, dependable, and suitable for their intended use [18,28]. Taken together, iBox allows clinicians to personalize patient follow-up and could potentially accelerate the development of new immunosuppressive treatments. The iBox Scoring System can be utilized to show how a new immunosuppressive therapy is better than the standard treatment from 6 to 24 months after transplantation in pivotal or investigative drug therapy studies.

OrganPredict: The OrganPredict (Precision and Reliability in Estimating Donor Immunological Compatibility for Transplantation) platform includes two major predictive models: L-TOP (Living-Donor Kidney Transplant Outcome Prediction) and D-TOP (Deceased-Donor Kidney Transplant Outcome Prediction) based on the UNOS database [29,30]. These models are used to optimize the matching process between donors and recipients in kidney transplants, predicting outcomes for both living and deceased donors. The predictions cover aspects like graft survival for up to 14 years. The authors utilized a deep Cox mixture model for prediction and compared it with the classical kidney donor profile index (KDPI). The results demonstrated that AI-based L-TOP and D-TOP outperformed the KDPI in evaluating transplant pairs based on graft survival, potentially enhancing deceased donor selection [29,30].

NephroCage: The NephroCage consortium began as a key project with support from the governments of Germany and Canada. It aimed to merge medical and technical knowledge to create and assess a practical example demonstrating the benefits of artificial intelligence (AI) in a specific medical scenario from nephrology. The NephroCage dataset consists of more than 8000 transplant patient cases over the past two decades with an average age of 51.7 years. NephroCage consortium members aim to prevent the occurrence of adverse post-transplant endpoints, such as loss of function, graft failure, and patient death, and also develop an open-source command line client for anonymization of HLA data of recipients and donors [31]. Of note, the NephroCage project is not finished yet, and it is difficult to judge from the available data.

In sum, the above-mentioned tools exemplify how AI can enhance decision-making in transplantation, from improving patient outcomes to optimizing organ allocation. iBox focuses more on post-transplant monitoring and prediction of long-term outcomes, while OrganPredict aids in both pre-transplant decision-making processes and post-transplant outcomes. Each tool leverages AI to handle complex datasets and improve the accuracy of predictions in the field of kidney transplantation.

iChoose Kidney: iChoose Kidney is designed to offer personalized prognosis estimates for patients with kidney disease, helping them understand the mortality risks associated with choosing between dialysis and kidney transplantation from living or deceased donors [32]. The mortality risk estimates provided by iChoose Kidney draw on data from the United States Renal Data System (2005–2015). This national database encompasses nearly all U.S. patients with end-stage kidney disease during that period, tracking outcomes related to transplantation and mortality. The personalized risk assessments are produced and confirmed through multivariable logistic regression models. These assessments provide mortality risks tailored to each patient’s age, race, and comorbid conditions, based on the information submitted in the patient information form. The iChoose Kidney model’s initial validation cohorts demonstrated a moderate ability to discriminate 3-year mortality risks, with a c-statistic of 0.7047 (95% confidence interval: 0.7029–0.7065) for dialysis patients and 0.7015 (95% confidence interval: 0.6875–0.7155) for transplant recipients [32]. Subsequent researchers revised the iChoose Kidney tool, and the coefficients from the updated version of the iChoose Kidney shared decision aid were comparable to the earlier version, demonstrating similar or enhanced predictive capabilities [33]. By incorporating a more detailed classification of time spent on dialysis and differentiating between preemptive transplants and other types, the predictive models can account for more subtleties in patient survival outcomes. Additionally, recognizing different dialysis methods—such as in-center hemodialysis, home hemodialysis, and peritoneal dialysis—helps to acknowledge the unique survival rates associated with each modality. This tool is designed as a collaborative decision-making aid for healthcare providers—including family doctors, nephrologists, transplant surgeons, nurses, social workers, and patient educators—to use with patients who are considered potential candidates for transplantation. It provides mortality risk estimates specifically for patients suffering from advanced chronic kidney disease or end-stage kidney disease (estimated glomerular filtration rate < 15 mL/min/1.73 m^2^). Legally, physicians must inform patients with end-stage kidney disease about all treatment options, including transplantation. Discussions about treatment choices for these patients should take place in chronic kidney disease clinics before dialysis begins, at the start of long-term dialysis, and throughout the transplant evaluation process. At the same time, iChoose Kidney is a perfect tool for patients with CKD or ESRD and enables easy access to learn the benefits of kidney transplantation over hemodialysis based on real-world data [34].

### 4.2. Heart Transplantation

Several AI-based tools are currently being employed and developed to predict outcomes in heart transplant patients, enhancing both the clinical decision-making process and patient management.

CARE (Cardiac Allograft Rejection Evaluator) Model: Arabyarmohammadi and colleagues developed a novel method for automated, comprehensive analysis of heart biopsy images called the Cardiac Allograft Rejection Evaluator (CARE). The authors utilized a total of 2900 endomyocardial biopsy images labeled with rejection grades (high versus low) and clinical trajectories (evident versus silent rejection). Using an image analysis approach, 370 quantitative morphology features describing lymphocytes and stroma were extracted from each slide. Two models were built to compare the subset of features associated with rejection grades to those linked with clinical trajectories. A proof-of-concept machine learning pipeline, the CARE, was then developed to test the feasibility of identifying the clinical severity of a rejection event. CARE extracts feature related to the shape, texture, and spatial architecture of muscle cells, immune cells, and stromal fibers in heart tissue specimens to predict rejection outcomes in heart transplant patients. The CARE model utilizes intuitive and explainable image features, making it more transparent and easier for clinicians to use compared to more opaque ‘black box’ AI models. The histopathological findings related to conventional rejection grades differ significantly from those associated with clinically evident allograft injury. Taken together, the quantitative assessment of a small set of well-defined morphological features can more accurately reflect the severity of rejection compared to the International Society of Heart and Lung Transplantation grades.

AI-ECG: In current clinical practice at some centers, heart transplant recipients undergo multiple endomyocardial biopsies (EMBs) every 1–4 weeks during the first year after transplantation, resulting in each patient potentially having up to 14 or more biopsies [35,36,37]. This frequent screening for allograft rejection is not only inconvenient due to the numerous hospital visits and associated costs but also carries risks of complications [38]. These complications can range from mild, such as pericardial effusion, to severe, like significant tricuspid valve regurgitation necessitating repeat cardiac surgery [38]. Therefore, it is crucial to provide heart transplant patients with safer, non-invasive screening methods for cardiac allograft rejection. Developing innovative diagnostic tools that enable remote screening could enhance our ability to monitor patients more frequently and reduce their need for constant visits to the transplant center for comprehensive care. These tools use electrocardiogram data to detect acute cellular rejection (ACR) among heart transplant recipients. AI-enhanced ECG demonstrated its ability to detect acute cellular rejection (ACR) with an area under the receiver operating characteristic (ROC) curve (AUC) of 0.84, within a 95% confidence interval (CI) of 0.78 to 0.90, and exhibited a sensitivity of 95% (19 out of 20 cases; 95% CI: 75–100%). In a prospective proof-of-concept screening study involving 56 participants and 97 ECG-biopsy pairs, AI-ECG identified ACR with an AUC of 0.78 (95% CI: 0.61–0.96) and achieved a sensitivity of 100% (2 out of 2 cases; 95% CI: 16–100%) [39]. To summarize, the findings reveal that a deep learning model utilizing 12-lead ECG can successfully identify moderate-to-severe acute cellular rejection (ACR) in heart transplant recipients. A proof-of-concept prospective analysis yielded promising results, however, needs verification in larger sample sizes. This study contributes to the expanding body of evidence supporting the potential of artificial intelligence models to improve cardiovascular care [33].

Machine Learning for Long-term Predictions: Research has been conducted on using machine learning models to predict 5-year mortality and graft failure in heart transplant recipients [40]. The study leveraged data from the International Society of Heart and Lung Transplant (ISHLT) registry to study 15,236 patients who received orthotopic heart transplants (OHT) between January 2005 and December 2009. A total of 342 variables were extracted and utilized to create a risk prediction model using a gradient-boosted machine (GBM) algorithm, aiming to predict the risk of graft failure and mortality five years after hospital discharge [40]. The authors excluded variables missing at least 50% of the observations and filled them using an AI model, which increased false predictions in their model. Five years after orthotopic heart transplantation (OHT), the mortality rate was 27.3% (*n* = 4161), while the graft failure (GF) rate was 28.1% (*n* = 4276) [40]. This might indicate that classical care and prediction methods after OHT are not as effective as desired, and further research is needed to improve post-transplant outcomes. The AUC for predicting 5-year mortality was 0.717 (95% CI 0.696–0.737), and for predicting graft failure, it was 0.716 (95% CI 0.696–0.736). The authors concluded that factors with the greatest impact on predicting 5-year mortality and graft failure were length of hospital stay, ages of the recipient and donor, body mass index of both the recipient and donor, and ischemic time. These tools represent a significant advancement in the field of transplant medicine, offering the potential for better patient outcomes through more precise and timely predictions. As these technologies continue to evolve, they are expected to become integral parts of clinical workflows in heart transplant centers soon.

### 4.3. Liver Transplantation

There are several AI-based tools and techniques used in the prediction of outcomes related to liver transplantation, ranging from academic research models to potentially commercially available tools. Here is a comparison of some notable examples:

Graft Failure Prediction: The collection of pre- and postoperative clinical data from both donors and recipients forms essential, non-invasive sources for machine learning to forecast graft failures. In this context, clinical and laboratory data analyzed using artificial neural networks (ANNs) more accurately predicted the risk of acute graft rejection compared to logistic regression (LR) at all evaluated time intervals [41]. In related research, pre-operative data were merged with donor and recipient characteristics, and the predictions from random forests (RF) and ANNs were strongly linked to the outcomes of grafts within 30 days [42]. The accuracy was further enhanced when RF incorporated validated elements from established predictive frameworks such as the Donor Risk Index (DRI) and Model for End-Stage Liver Disease (MELD) [42]. This study illustrates that not only is machine learning more precise than conventional models in predicting, but the selection of parameters is also crucial for its predictive efficacy.

Machine Learning Models vs. Traditional Scoring Systems in Liver Transplantation: Most recently, Chongo et al. performed a systematic analysis to assess the utility of ML models in prognostication for LT, comparing their performance and reliability to established traditional scoring systems [43]. In all the research reviewed, machine learning (ML) algorithms and models were constructed using variables from both donors and recipients prior to transplantation. Predictions of short-term mortality typically covered up to 90 days, whereas long-term predictions extended up to five years. The analysis of the area under the receiver operating characteristic (AUROC) curve indicated that ML models consistently provided satisfactory to excellent accuracy in predicting both short- and long-term mortality, as well as the risk of post-transplant complications in liver transplant patients [43]. Additionally, AUROC analysis showed that ML models surpassed traditional models and scoring systems, including widely recognized models such as the Model for End-Stage Liver Disease (MELD), Delta MELD (D-MELD), Survival Outcomes Following Liver Transplantation (SOFT), Pediatric SOFT (P-SOFT), Balance of Risk (BAR), Donor Risk Index (DRI), Age-Bilirubin-INR-Creatinine (ABIC) score, Chronic Liver Failure Consortium Organ Failure score (CLIF-C OFs), Acute-on-Chronic Liver Failure score (CLIF-C ACLFs), and the Sequential Organ Failure Assessment (CLIF SOFA) [12]. Moreover, ML models also demonstrated advantages over models based on Cox proportional hazards and logistic regression [43]. In evaluating 90-day mortality predictions, the Random Forest (RF) model showcased the highest area under the curve (AUC) score of 0.940, outperforming other machine learning models [43]. From the six studies reviewed that explored the use of machine learning models to predict post-liver transplant complications, an analysis revealed that the ‘gradient boosting machine’ model excelled above others in forecasting the likelihood of complications such as graft-versus-host disease (GVHD), pneumonia, and acute kidney injury (AKI) [12]. Conversely, the RF model was more effective in accurately predicting the risks of sepsis and AKI following liver transplantation [43]. Strong evidence shows that machine learning models are capable of significantly improving decision-making processes in allograft allocation and liver transplantation, representing a major advancement in the area of prognostication. The progress in this field suggests that more robust and widely applicable AI tools could soon be on the horizon, enhancing both the efficiency and outcomes of liver transplantation programs [16].

## 5. Ethical Aspects of AI Tools in Organ Transplantation

The integration of AI tools into the field of organ transplantation represents a significant shift, with the potential to impact various aspects of the overall transplantation process for both patients and physicians. As previously discussed, AI is being applied to improve organ matching, predict post-transplant outcomes, and enhance the efficiency of transplant logistics. However, these advancements bring important ethical considerations that need to be addressed to ensure that AI tools are used responsibly and equitably [44]. This section explores the key ethical challenges associated with the use of AI in transplantation, including issues related to bias, fairness, transparency, accountability, patient autonomy, and the broader implications for clinical workflows and healthcare systems.

### 5.1. Promoting Equity by Addressing Bias and Fairness in AI Systems

AI systems in transplantation, like in other areas of healthcare, are susceptible to biases that may perpetuate or even exacerbate existing disparities. These biases can stem from the data used to train AI algorithms, which often reflect historical inequities in healthcare access and outcomes [45]. For example, studies have shown that AI systems can exhibit racial bias, potentially leading to discriminatory practices in organ allocation. The implications of such biases are particularly severe in transplantation, where the stakes involve life and death decisions [46,47]. Efforts to mitigate bias involve developing more representative datasets and implementing algorithmic fairness techniques [45,48]. However, these measures alone may not fully eliminate the risk of unintended consequences. Therefore, continuous monitoring for bias and integrating bias detection tools and techniques, such as fairness audits and bias impact assessments, into the AI development and deployment processes is necessary [49].

Moreover, balancing fairness with accuracy is a critical challenge. While creating fair AI systems is paramount, it should not come at the cost of compromising the system’s accuracy and efficiency, particularly in high-stakes fields like organ transplantation [50]. A recently published article by Audrey Lebret discusses fairness in AI in the context of healthcare, emphasizing the need to mitigate bias and ensure equitable outcomes [51]. Here, the author discusses that the European human rights law approach directly supports these objectives by providing a legal and ethical framework that demands fairness, non-discrimination, and respect for human dignity in all AI applications. For instance, the article’s focus on bias in AI and its impact on healthcare aligns with the European human rights law’s emphasis on non-discrimination and the right to health (as articulated in Article 35 of the EU Charter). Moreover, the call for transparency and accountability in AI systems resonates with the European legal principles that require clear, explainable, and accountable decision-making processes. In addition to these technical and ethical challenges in bias and fairness, there is a broader need for stakeholder engagement, including input from AI developers, clinicians, and patient advocacy groups, to promote equity in AI-driven transplantation [52]. Organizations and institutions must take an active role in setting standards for fairness and enforcing compliance, while also educating end users about the potential biases in AI systems. This comprehensive approach is necessary to ensure that AI systems contribute to a more equitable healthcare system, particularly in the critical domain of organ transplantation [53].

### 5.2. Transparency and Explainability: Unveiling the Black Box Through Transparency and Explainability

The rapid advancement of AI technologies, particularly in fields like transplantation, offers tremendous potential to improve patient outcomes. However, the complexity of AI algorithms, especially those based on deep learning, presents significant challenges related to transparency and explainability. In high-stakes contexts like organ transplantation, where decisions directly impact life and death, it is essential that healthcare providers and patients understand how AI-generated recommendations are made.

#### 5.2.1. Transparency and Its Ethical Importance

Transparency in AI refers to the openness and clarity with which the processes and operations of AI systems can be understood by humans [54]. This encompasses the data used to train AI algorithms, the architecture of the models, and the logic behind specific outputs. In the realm of transplantation, ensuring transparency is essential for several reasons:

1. Data Transparency: The datasets used to train AI systems must be accessible, representative, and of high quality. This involves disclosing the sources of data, the methods of data collection, and addressing any potential biases. In transplantation, where patient outcomes can vary widely, transparent data practices help stakeholders understand the factors influencing AI recommendations [55].
2. Algorithmic Transparency: The complexity of AI models, particularly deep learning algorithms, often results in what is termed a “black box” effect, where the internal workings of the model are opaque [56]. While it may not always be feasible to disclose every detail of the model’s operations, providing a high-level overview of how the model functions and the types of patterns it detects is crucial. This allows clinicians to better understand and trust AI-assisted decisions.

#### 5.2.2. Provide Explainability to Bridge the Gap Between Complexity and Comprehension

Explainability in AI refers to the ability of the system to provide understandable reasons for its decisions. While transparency ensures that the processes are open, explainability makes these processes comprehensible to those who rely on AI outputs, such as clinicians and patients.

1. Interpretable Models: One approach to enhancing explainability is using simpler, more interpretable models like decision trees or linear regression, which offer straightforward explanations. However, in complex domains like transplantation, where numerous factors influence outcomes, these simpler models might not be sufficiently accurate [57]. The challenge lies in balancing the need for interpretability with the accuracy required for clinical decision-making [58].
2. Post-Hoc Explainability Tools: Techniques such as Local Interpretable Model-agnostic Explanations (LIME) [59] and SHapley Additive exPlanations (SHAP) [60] have been developed to provide post-hoc explanations for complex AI models. These tools allow clinicians to see how different features contributed to the AI’s decisions, offering insights that can increase confidence in AI-generated recommendations.
3. Contextual Explanations: Tailoring explanations to the audience is essential. For clinicians, detailed explanations that align with medical reasoning are necessary, while for patients, explanations should focus on the implications for their health and treatment options [61,62]. Providing relevant and understandable information helps ensure that both patients and healthcare providers can make informed decisions based on AI outputs.

#### 5.2.3. The Ethical Imperative of Transparency and Explainability

Ensuring that AI systems in transplantation are both transparent and explainable is not merely a technical challenge but an ethical imperative [63]. The use of AI in healthcare must adhere to core ethical principles, including:

1. Autonomy and Informed Consent: Patients have the right to make informed decisions about their care, which requires a clear understanding of AI recommendations. Explainable AI respects patient autonomy by enabling informed choices based on a full understanding of the risks and benefits. Informed consent is a foundational principle of medical ethics, requiring that patients understand and agree to the treatments they receive. The use of AI in transplantation introduces new complexities to the process of obtaining informed consent. Patients may have a limited understanding of AI technology and its role in their care, which can hinder their ability to make fully informed decisions. This is particularly concerning when AI is used to predict outcomes or recommend specific treatments, as patients may not be aware of the limitations or uncertainties inherent in these tools. Healthcare providers must ensure that patients are adequately informed about the use of AI in their care, including the potential risks and benefits. This involves not only explaining how AI is used but also discussing the limitations of the technology and the potential for error. Ethical frameworks for informed consent in the era of AI must evolve to address these new challenges, ensuring that patients retain autonomy over their healthcare decisions [64].
2. Non-Maleficence and Beneficence: Transparent and explainable AI systems help prevent harm by allowing for the identification and correction of errors or biases. This ensures that AI tools contribute positively to patient outcomes. While AI can provide valuable insights and support to healthcare providers, there is a risk that over-reliance on these tools could undermine the autonomy of both physicians and patients. Ethical concerns arise when AI recommendations are followed without critical evaluation or when they conflict with the clinical judgment of physicians [65]. The concept of “algorithmic authority” refers to the growing reliance on AI systems to make decisions traditionally made by humans. In transplantation, this could lead to situations where AI-driven decisions override human judgment, potentially leading to outcomes that are not in the best interest of the patient. To address these concerns, it is essential to establish clear guidelines that balance the use of AI with human oversight, ensuring that AI tools augment rather than replace clinical expertise [66,67].
3. Justice: Transparency and explainability are critical for identifying and addressing potential biases in AI systems. Ensuring fairness and equity in AI applications is essential, especially in transplantation, where disparities in access and outcomes must be carefully managed. The adoption of AI in transplantation has the potential to exacerbate existing inequalities in healthcare access. Advanced AI tools are often developed and implemented in well-resourced healthcare settings, potentially leaving less affluent institutions and their patients at a disadvantage. This raises ethical questions about the equitable distribution of the benefits of AI, particularly in a field as critical as transplantation, where access to care can be a matter of life and death [68]. Efforts to promote equity in AI deployment include the development of policies and frameworks that ensure fair access to AI technologies, regardless of geographic location or socioeconomic status. Additionally, there is a need for global collaboration to address disparities in AI development and implementation, ensuring that the benefits of AI in transplantation are available to all patients [69,70].
4. Safety and Privacy: The deployment of AI in transplantation relies on the analysis of large datasets, including sensitive patient information. This raises significant ethical concerns regarding data privacy and security. The potential for data breaches, unauthorized access, and misuse of patient information is a major risk that must be carefully managed. Moreover, the sharing of data across institutions and international borders for AI development and validation purposes introduces additional layers of complexity and risk [71]. Ethical guidelines for AI in healthcare emphasize the importance of robust data protection measures, including encryption, anonymization, and strict access controls. However, the dynamic nature of AI development, which often requires continuous data collection and analysis, poses ongoing challenges to maintaining patient privacy. Ensuring that patient data is used responsibly and securely is essential to maintaining public trust and safeguarding patient rights [72].

In summary, to create ethically responsible AI applications in transplantation and other areas of healthcare, transparency and explainability must be prioritized. This involves not only implementing technical solutions but also committing to ethical standards that prioritize patient safety, trust, and fairness. By embedding these principles into AI development and deployment, AI systems can be developed that improve, rather than undermine, the quality and equity of healthcare.

## 6. Discussion

### 6.1. Impact on Patient–Physician Relationships

The introduction of AI into the transplantation process can also affect the traditional patient–physician relationship [73]. Trust is a key component of this relationship, and the use of AI tools that patients do not fully understand can undermine this trust. Patients may feel disconnected from their physicians if they believe that critical decisions about their care are being made by algorithms rather than human judgment. Moreover, the perception that decisions are being made by machines rather than humans can lead to skepticism and concern, potentially fostering a sense of depersonalization in what is already a high-stress medical environment [74].

To maintain a strong patient–physician relationship, it is crucial that healthcare providers clearly communicate the role of AI in the decision-making process and follow AI-reporting guidelines [75]. This includes explaining how AI complements and supports, rather than replaces, the expertise of the physician. Physicians should emphasize that they remain at the center of the decision-making process, using AI as a tool to enhance their judgment rather than as a substitute for their clinical experience. Additionally, healthcare providers should reassure patients that AI systems are designed to reduce risks and improve outcomes so that their personalized care remains the highest priority.

Fostering an open and transparent dialogue is essential to overcoming the potential negative effects of AI on the patient–physician relationship. It involves not only explaining how AI works but also addressing any concerns or misconceptions patients may have about the technology [76]. By encouraging patients to ask questions and providing clear, accessible explanations, physicians may improve the acceptance of dedicated AI tools and build higher trust in their use [77]. In doing so, healthcare providers can help ensure that patients remain active participants in their treatment, even as AI plays an increasingly prominent role in transplantation [78,79].

### 6.2. Is AI Changing the Clinical Workflows in Organ Transplantation?

The integration of AI into clinical workflows is set to significantly alter the role of physicians in transplantation and other medical fields. While AI brings powerful tools that support decision-making, its presence will introduce new complexities and may reshape traditional practices. One of the most notable changes is probably the influence on decision-making. AI’s ability to process vast amounts of data and generate predictive insights can improve diagnosis, organ matching, and post-transplant care. Physicians will be faced with assessing and interpreting these recommendations while ensuring they align with clinical judgment and patient needs. Thus, AI could offer the potential to shift the physician’s focus from time-consuming data analysis to more patient-centered care [80]. By automating administrative tasks such as reviewing patient histories and assessing organ matches, AI can free up physicians’ time, allowing them to dedicate more attention to patient interactions and treatment planning. However, this requires physicians to adapt to a model where they are more reliant on AI to process data while still maintaining oversight of critical decisions. Here, physicians may find themselves in situations where AI recommendations conflict with their own clinical judgment [81]. In cases where AI-driven decisions lead to adverse outcomes, determining responsibility, whether it lies with the physician, the healthcare provider, or the developers of the AI system, will be a critical issue. New legal frameworks and professional guidelines need to emerge to clarify physicians’ responsibilities when using AI tools.

Moreover, AI will likely lead to changes in time management. While certain tasks may be automated, such as data entry and scheduling, physicians may need to dedicate more time to reviewing AI-generated recommendations or explaining these insights to patients [82]. This shift in workload distribution will require adjustments in how physicians manage their time, with a greater emphasis on complex decision-making and patient communication [83].

Finally, as AI becomes a more embedded part of clinical workflows, physicians, as the primary domain experts, will be required to take on the responsibility of continuous monitoring and quality control. AI systems must be regularly assessed to ensure they remain accurate, unbiased, and aligned with the latest medical knowledge [84]. Physicians will need to collaborate with AI specialists to adjust and update AI algorithms as needed, ensuring that these tools evolve alongside advancements in medical science and changes in patient populations.

### 6.3. Future Perspectives

In the future, the role of AI in solid organ transplantation is expected to expand, with continuous advancements in algorithm development, data integration, and personalized medicine. Future research should focus on enhancing the interpretability and transparency of AI models, addressing ethical concerns, and ensuring the equitable distribution of AI benefits across diverse patient populations. Collaboration between AI specialists, computer scientists, clinicians, ethicists, and policymakers will be essential in shaping the future of AI in transplantation. This interdisciplinary approach will help in developing more robust guidelines that govern the use of AI, ensuring that it is applied in ways that maximize patient benefit while minimizing potential risks. Moreover, the future may see the integration of AI with other emerging technologies such as genomics, proteomics, and telemedicine, further pushing the boundaries of personalized care in transplantation. The application of ML models demonstrates significant potential in fields such as lung transplantation [85,86], where AI is being investigated to enhance transplantation outcomes. Nevertheless, the development of robust ML- and DL-based algorithms for accurately predicting graft failures in lung and pancreas transplantations necessitates larger datasets with greater ethnic diversity. Recent studies further highlight the growing role of machine learning in predicting and improving patient outcomes, particularly in kidney transplantation, reflecting a broader trend toward the integration of AI technologies across various organ transplant procedures. By continuing to innovate and address the challenges associated with AI, the transplantation community can leverage these tools to achieve better patient outcomes and contribute to the evolution of organ transplantation as a whole.

## 7. Conclusions

With the progression of data analysis techniques, moving from traditional statistical methods to the use of advanced AI models, there has been a remarkable improvement in the precision and versatility of predictive tools. This shift has empowered clinicians to make more informed decisions, ultimately improving patient treatments. In kidney transplantation, tools like iBox, SOTAI, OrganPredict, NephroCage, and iChoose Kidney exemplify how AI can provide tailored predictions for graft survival and patient outcomes. These tools offer more personalized care plans, reducing the likelihood of rejection and enhancing the longevity of the transplanted organs. Similarly, in heart transplantation, models like CARE and AI-ECG have shown promising results in predicting rejection and guiding therapy adjustments. Liver transplantation has also benefited from AI’s capabilities, particularly in predicting graft failure and comparing the performance of machine learning models with traditional scoring systems. These advancements underline the superiority of AI-driven tools in dedicated prognostic models, thereby supporting more precise clinical interventions.

However, the integration of AI in transplantation is not without ethical challenges. The use of AI tools raises concerns regarding transparency, accountability, data privacy, and the potential for bias. These ethical aspects must be carefully considered to ensure that the deployment of AI technologies in transplantation upholds the highest standards of fairness and equity. As AI becomes more integrated into clinical workflows, the patient–physician relationship is being reshaped, requiring physicians to balance trust-building with AI transparency while adapting to new roles of oversight, technical proficiency, and collaborative decision-making to ensure the responsible use of AI in patient care.
